# Rabdosianone I, a Bitter Diterpene from an Oriental Herb, Suppresses Thymidylate Synthase Expression by Directly Binding to ANT2 and PHB2

**DOI:** 10.3390/cancers13050982

**Published:** 2021-02-26

**Authors:** Motoki Watanabe, Yasumasa Yamada, Yoichi Kurumida, Tomoshi Kameda, Mamiko Sukeno, Mahiro Iizuka-Ohashi, Yoshihiro Sowa, Yosuke Iizumi, Hideki Takakura, Shingo Miyamoto, Toshiyuki Sakai, Michihiro Mutoh

**Affiliations:** 1 Department of Molecular-Targeting Prevention, Kyoto Prefectural University of Medicine, Kyoto 602-8566, Japan; iom0220@koto.kpu-m.ac.jp (M.I.-O.); ysowa@koto.kpu-m.ac.jp (Y.S.); yiizumi@koto.kpu-m.ac.jp (Y.I.); takakura@koto.kpu-m.ac.jp (H.T.); mimutoh@koto.kpu-m.ac.jp (M.M.); 2 Department of Food Science and Human Nutrition, Baika Women’s University, Osaka 567-8578, Japan; y-yamada@baika.ac.jp; 3 Artificial Intelligence Research Center, National Institute of Advanced Industrial Science and Technology (AIST), Tokyo 135-0064, Japan; yoichi.kurumida@aist.go.jp (Y.K.); kameda-tomoshi@aist.go.jp (T.K.); 4 Drug Discovery Center, Kyoto Prefectural University of Medicine, Kyoto 602-8566, Japan; sukeno@koto.kpu-m.ac.jp (M.S.); tsakai@koto.kpu-m.ac.jp (T.S.); 5 Department of Endocrine and Breast Surgery, Kyoto Prefectural University of Medicine, Kyoto 602-8566, Japan; 6 Epidemiology and Prevention Division, Center for Public Health Sciences, National Cancer Center, Tokyo 104-0045, Japan; miyamoto.shingo@otsuka.jp

**Keywords:** natural products, rabdosianone I, thymidylate synthase, adenine nucleotide translocase 2, prohibitin 2, chemical biology

## Abstract

**Simple Summary:**

In the present study, we found the novel pleiotropic regulation of the oncogene product thymidylate synthase (TS) by a chemical biology approach to identify rabdosianone I-binding proteins. Rabdosianone I, which is extracted from a traditional Asian herb *Isodon japonicus* Hara for longevity, suppressed TS expression at mRNA and protein levels. We immobilized rabdosianone I onto nano-magnetic beads and identified two mitochondrial proteins, adenine nucleotide translocase 2 (ANT2) and prohibitin 2 (PHB2), as the direct targets of rabdosianone I in cancer cells. Mechanistically, the knockdown of ANT2 or PHB2 promoted proteasomal degradation of the TS protein. In addition, PHB2 reduced TS mRNA levels. Thus, we provide previously unknown mechanisms of TS regulation by ANT2 and PHB2 and propose the possibility of rabdosianone I as a promising lead compound for the discovery of a novel TS suppressor.

**Abstract:**

Natural products have numerous bioactivities and are expected to be a resource for potent drugs. However, their direct targets in cells often remain unclear. We found that rabdosianone I, which is a bitter diterpene from an oriental herb for longevity, *Isodon japonicus* Hara, markedly inhibited the growth of human colorectal cancer cells by downregulating the expression of thymidylate synthase (TS). Next, using rabdosianone I-immobilized nano-magnetic beads, we identified two mitochondrial inner membrane proteins, adenine nucleotide translocase 2 (ANT2) and prohibitin 2 (PHB2), as direct targets of rabdosianone I. Consistent with the action of rabdosianone I, the depletion of ANT2 or PHB2 reduced TS expression in a different manner. The knockdown of ANT2 or PHB2 promoted proteasomal degradation of TS protein, whereas that of not ANT2 but PHB2 reduced TS mRNA levels. Thus, our study reveals the ANT2- and PHB2-mediated pleiotropic regulation of TS expression and demonstrates the possibility of rabdosianone I as a lead compound of TS suppressor.

## 1. Introduction

Natural products have been a rich repository of medical supplies [1]. In particular, plant extracts from many types of herbs have been used throughout history. For example, according to legend, Kukai (774–835), one of the most well-known Japanese monks, treated patients using the traditional herb *Isodon japonicus* Hara during the Heian period in Japan. *Isodon japonicus* Hara, known as Enmeiso, which means “grass of longevity” in Japanese, has been infused to make tea for health purposes. However, how *Isodon japonicus* Hara may contribute to disease prevention and treatment, including anticancer activity, is not well characterized.

It was reported that several types of bitter diterpenes (e.g., oridonin, enmein, and rabdosianone I) were extracted from *Isodon japonicus* Hara [2]. A number of papers have reported that oridonin inhibited malignant behaviors of cancer [3]. Enmein promoted the survival rate of the Ehrlich ascites carcinoma tumor model [4]. However, to date, no attempt has been made to demonstrate the anticancer activity of rabdosianone I. 

In this study, we first aimed to investigate whether rabdosianone I shows anticancer effects using human colorectal cancer cell lines. We found that rabdosianone I suppressed cell growth by reducing the oncogene product thymidylate synthase (TS) at mRNA and protein levels. TS is an S-phase enzyme that catalyzes the reductive methylation of dUMP to produce the de novo source of thymidylate, a precursor of DNA synthesis [5]. TS functions as an oncogene [6], and a number of reports have demonstrated its expression to be related to the prognosis of colorectal cancer [7,8,9,10] and sensitivity to TS-targeted chemotherapy such as 5-fluorouracil [11,12,13,14,15,16]. 

Furthermore, in order to investigate the mechanism(s) of rabdosianone I-mediated TS reduction, we immobilized rabdosianone I onto nano-magnetic beads and identified its two direct targets: Adenine nucleotide translocase 2 (ANT2) and prohibitin 2 (PHB2), both of which are known as mitochondrial inner membrane proteins [17]. ANT2 has been reported to be expressed in proliferative cells, including cancer cells [18,19], and function as an oncogenic protein [20,21]. Likewise, PHB2, which physiologically forms a complex with ANT2 [17], has pleiotropic functions to regulate cell survival, cellular metabolism, aging, and inflammation [22]. PHB2 is also implicated in cancer progression [22,23]. In this study, we found that the deletion of ANT2 or PHB2 resulted in the downregulation of TS at mRNA and protein levels, which is analogous to the mode of action of rabdosianone I. 

Thus, our study clarified a previously unknown mechanism of an ancient herbal remedy, i.e., pleiotropic regulation of TS, and may provide a starting point for the design of novel TS suppressors.

## 2. Results

### 2.1. Rabdosianone I Inhibits Cell Growth by Arresting Cells in S Phase

**Figure 1:**
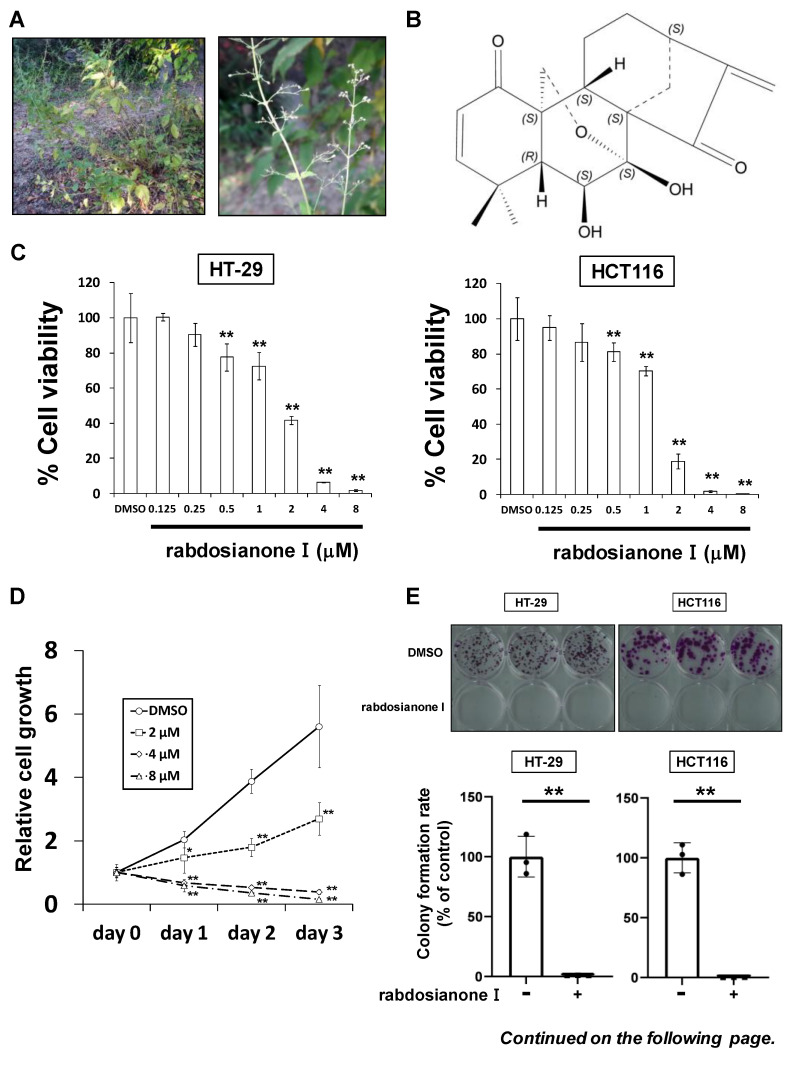

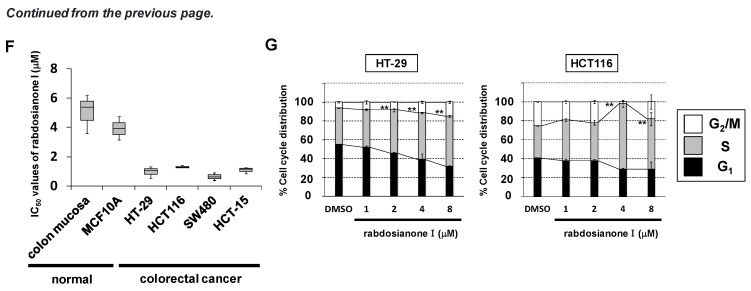
Rabdosianone I inhibits cell growth by inducing S phase arrest. (**A**) Distant view (left panel) and close-range view (right panel) of *Isodon japonicus* Hara. (**B**) Chemical structure of rabdosianone I. (**C**) The dose-dependency of the growth inhibitory effect of rabdosianone I. HT-29 and HCT116 cells were treated with rabdosianone I at the indicated concentrations for 24 h. Cell viability was measured using a Cell Counting Kit-8 assay at the indicated times. Results with DMSO (dimethyl sulfoxide) were taken as 100%. Columns, means (*n* = 3); bars, SD. ** *p* < 0.01, significantly different from the DMSO-treated control. (**D**) The time dependency of the growth inhibitory effect of rabdosianone I. HT-29 cells were treated with rabdosianone I at the indicated concentrations for 24 h. Cell viability was measured using a Cell Counting Kit-8 assay at the indicated times. Each result of OD450 nm value with day 0 (treatment day) were taken as 1. Points, means (*n* = 3); bars, SD. * *p* < 0.05, ** *p* < 0.01, significantly different from the DMSO-treated control. (**E**) Effects of rabdosianone I on colony formation. HT-29 and HCT116 cells were treated with rabdosianone I at 4 μM. After further incubation, colonies were fixed and stained by crystal violet. The images of stained colonies are shown (upper panel). Colony formation rate is shown in the graph (lower panel). Columns, means (*n* = 3); bars, SD, ** *p* < 0.01, significantly different from the DMSO-treated control. (**F**) The difference of IC_50_ values of rabdosianone I between non-cancerous and cancer cell lines. The growth inhibition curve of cells treated with rabdosianone I for 72 h was determined using a Cell Counting Kit-8 assay. The IC_50_ values of rabdosianone I in non-cancerous cell lines (mouse colon mucosa cells and MCF-10A cells) and human colorectal cancer cell lines (HT-29, HCT116, SW480, and HCT-15) were obtained from each growth inhibition curve. The grouped data of IC_50_ values from three independent experiments are shown as box plots. The median value is shown by a horizontal line in the box plot. (**G**) Cell cycle analysis following rabdosianone I treatment. HT-29 and HCT116 cells were treated with rabdosianone I at the indicated concentrations for 72 h. The DNA content of the cells was determined by flow cytometry. The percentages of cells in the G_1_, S, and G_2_/M phases of the cell cycle are shown. Columns, means (*n* = 3); bars, SD. ** *p* < 0.01, significantly different from the S phase distribution of the DMSO-treated control.

Rabdosianone I is a bitter diterpene isolated from an oriental herb, *Isodon japonicus* Hara (Figure 1A). The chemical structure of rabdosianone I is shown in Figure 1B, and the purity analyses by LCMS, including LC chromatogram (Supplementary Figure S1A) and TOF-MS (Supplementary Figure S1B), estimated its purity to be approximately 98%. We first investigated the rabdosianone I-mediated effects on cell growth using human colorectal cancer cell lines. The treatment of rabdosianone I significantly suppressed the cell growth of HT-29 and HCT-116 cells in a dose-dependent manner (Figure 1C). Furthermore, rabdosianone I treatment at 4–8 μM exhibited cytotoxicity in a time-dependent manner (Figure 1D). Consistently, 4 μM rabdosianone I almost eliminated the colony formation of HT-29 and HCT116 cells (Figure 1E). Importantly, the IC_50_ values of rabdosianone I were relatively lower in human colorectal cancer cells (HT-29, HCT116, SW480, and HCT-15) than in non-cancerous cells (mouse colon mucosa cells and normal breast epithelial MCF-10A cells) (Figure 1F). In order to examine the mechanism of these growth inhibitory effects by rabdosianone I, we performed cell cycle analysis by flow cytometry and found that rabdosianone I induced significant S phase arrest at 4 or 8 μM in HT-29 and HCT116 cells (Figure 1G) with the induction of apoptosis (Supplementary Figure S2).

### 2.2. Rabdosianone I Suppresses Thymidylate Synthase Expression at mRNA and Protein Levels

**Figure 2:**
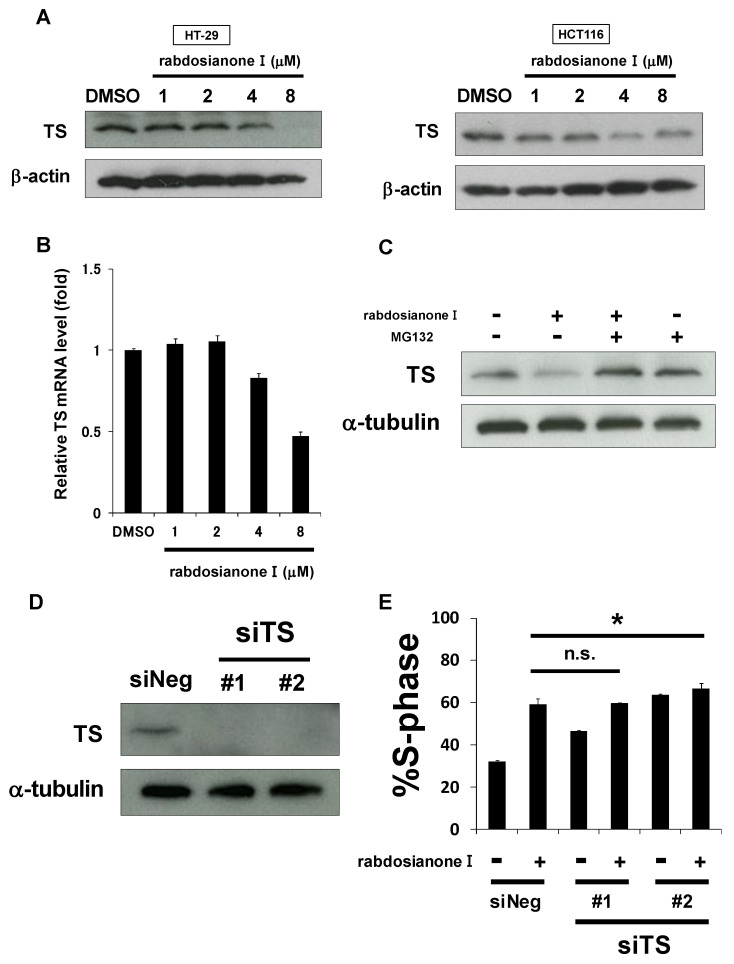
Rabdosianone I suppresses thymidylate synthase (TS) expression at mRNA and protein levels. (**A**) TS protein expression in rabdosianone I-treated cells. The expressions of TS protein were analyzed by Western blotting in HT-29 cells (left panel) and HCT116 cells (right panel) treated with rabdosianone I at the indicated concentrations for 48 h. β-Actin was used as a loading control. (**B**) TS mRNA expression in rabdosianone I-treated cells. The expression of TS mRNA was analyzed by quantitative PCR in HT-29 cells treated with rabdosianone I at the indicated concentrations for 48 h. TS mRNA was normalized to GAPDH mRNA, and the results with DMSO were taken as 1. Columns, means (*n* = 3); bars, SD. (**C**) TS protein expression in cells treated with rabdosianone I with or without MG132. The expression of TS protein was analyzed by Western blotting in HT-29 cells treated with 8 μM rabdosianone I with or without 10 μM MG132 for 48 h. α-Tubulin was used as a loading control. (**D**) Knockdown efficacy of two siRNAs (#1 and #2) targeting different sequences of the TS gene. The expression of TS protein was analyzed by Western blotting in HT-29 cells treated with siNeg or siTS for 48 h. α-Tubulin was used as a loading control. (**E**) Cell cycle analysis following rabdosianone I treatment in TS depleted cells. HT-29 cells were treated with rabdosianone I at 8 μM for 48 h with or without siTS transfection. The DNA content of the cells was measured by flow cytometry. The percentages of cells in S phase of the cell cycle are shown. Columns, means (*n* = 3); bars, SD. * *p* < 0.05, significantly different from rabdosianone I with siNeg-treated control.

As one of the most important molecules for entering the S phase is thymidylate synthase (TS), we hypothesized that rabdosianone I downregulates TS expression levels. As shown in Figure 2A (left panel), rabdosianone I at 4 or 8 μM reduced TS expression in HT-29 cells, which is consistent with S arrest induced by rabdosianone I (Figure 1G, left panel). In HCT116 cells, 4 μM rabdosianone I reduced TS expression (Figure 2A, right panel), consistent with the significant accumulation of cells in the S phase following treatment with 4 μM rabdosianone I (Figure 1G, right panel). However, the suppression of TS was recovered after the treatment of 8 μM rabdosianone I (Figure 2A, right panel), which implies that there may be some sort of feedback system in HCT116 cells. These results suggest that rabdosianone I-mediated TS reduction causes S phase arrest.

Next, we investigated the mechanism of rabdosianone I-mediated reduction of TS expression. First, we observed that 4 or 8 μM rabdosianone I reduced TS mRNA levels in HT-29 cells (Figure 2B), which is consistent with the Western blotting results shown in Figure 2A (left panel). Second, we also considered the possibility that rabdosianone I promotes proteasomal degradation of TS protein. As shown in Figure 2C, rabdosianone I-mediated reduction in TS protein was inhibited by the addition of the proteasomal inhibitor MG132. This suggests that rabdosianone I suppresses TS expression at both mRNA and protein levels.

To examine whether TS reduction is required to induce rabdosianone I-mediated S phase arrest, we performed genetic TS knockdown experiments using siRNA using two siRNAs targeting different sequences of the TS gene (Figure 2D). TS silencing further induced little or no S phase arrest in rabdosianone I-treated HT-29 cells (Figure 2E), suggesting that the reduction in TS expression is partially at least responsible for rabdosianone I-induced S phase arrest.

### 2.3. Rabdosianone I Directly Binds to the Mitochondrial Inner Proteins ANT2 and PHB2

**Figure 3:**
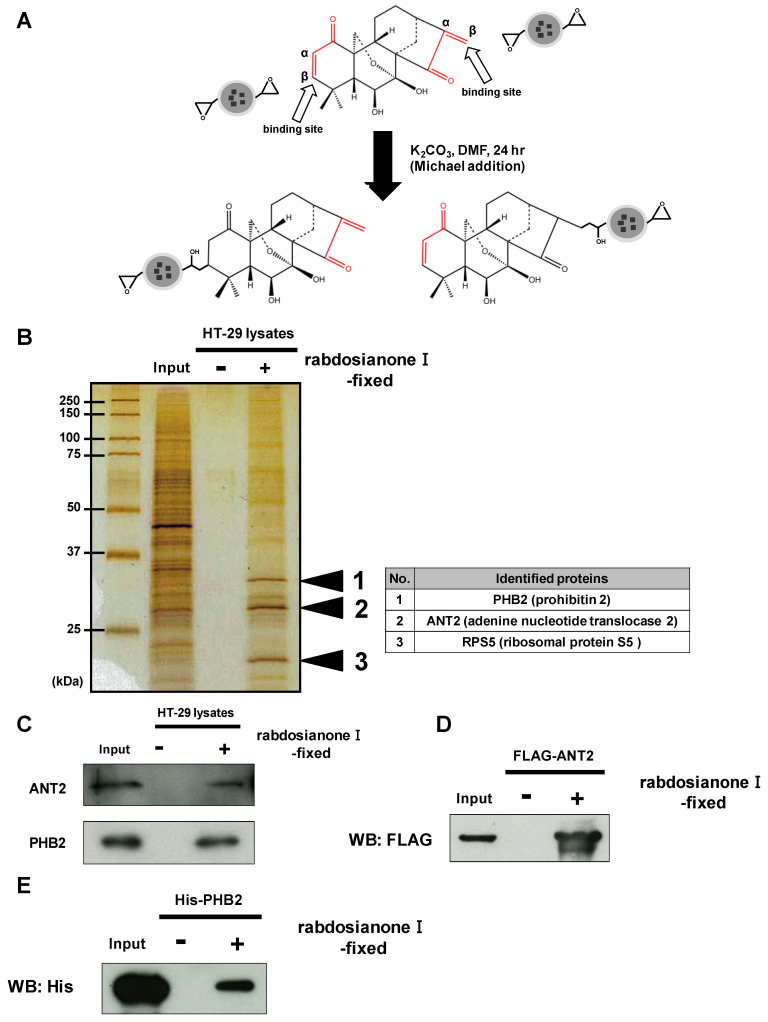
Rabdosianone I directly binds to ANT2 and PHB2. (**A**) Scheme of fixation of rabdosianone I onto magnetic FG beads and the two predicted structures of rabdosianone I-fixed beads. α, β-Unsaturated ketones are shown by the red lines. (**B**) Identification of rabdosianone I-binding proteins. Three rabdosianone I-binding proteins were purified from whole-cell extracts of HT-29 cells with rabdosianone I-fixed FG beads and detected by silver staining. Mass spectrometry analysis identified three rabdosianone I-binding proteins, as shown in the right table. (**C**) Confirmation of the interaction of rabdosianone I with ANT2 and PHB2. Bound ANT2 and PHB2 were detected by Western blotting with either anti-ANT2 or PHB2 antibody. (**D**) Confirmation of the interaction of rabdosianone I with recombinant ANT2. Purified recombinant FLAG-ANT2 was incubated with rabdosianone I-fixed FG beads, and bound FLAG-ANT2 was detected by Western blotting with the anti-FLAG antibody. (**E**) Confirmation of the interaction of rabdosianone I with recombinant PHB2. Purified recombinant His-PHB2 was incubated with rabdosianone I-fixed FG beads, and bound His-PHB2 was detected by Western blotting with the anti-His antibody.

To investigate the precise mechanism(s) of the anticancer activity of rabdosianone I, we examined rabdosianone I-binding proteins. Rabdosianone I has two α, β-unsaturated ketones (Figure 3A), which are reactive Michael acceptors. We then immobilized the α, β-unsaturated ketones of rabdosianone I on epoxy rings of nano-magnetic beads by Michael addition (Figure 3A). We next incubated rabdosianone I-immobilized beads with whole-cell extracts of HT-29 cells. Silver staining of purified rabdosianone I-binding proteins revealed three bands, which were identified by MALDI-TOF MS analysis as prohibitin 2 (PHB2), adenine nucleotide translocase 2 (ANT2), and ribosomal protein S5 (Figure 3B). Among these proteins, we focused on the inner mitochondrial membrane proteins ANT2 and PHB2 because both have been reported as oncogenic proteins [18,19,20,21]. We then confirmed the binding of ANT2 and PHB2 to rabdosianone I by Western blotting with the respective specific antibody (Figure 3C). Furthermore, we confirmed that purified recombinant FLAG-tagged ANT2 protein and His-tagged PHB2 protein bound to rabdosianone I-immobilized beads (Figure 3D,E). We then investigated whether direct bindings of rabdosianone I to ANT2 and PHB2 affects their expressions. The treatment of rabdosianone I showed no alteration of ANT2 (Supplementary Figure S3A) and PHB2 (Supplementary Figure S3B) protein levels. These results demonstrate that rabdosianone I directly binds to both ANT2 and PHB2 without alteration of expression of these proteins.

### 2.4. Molecular Binding Models Represent the Strong Interaction between Rabdosianone I with ANT2

**Figure 4:**
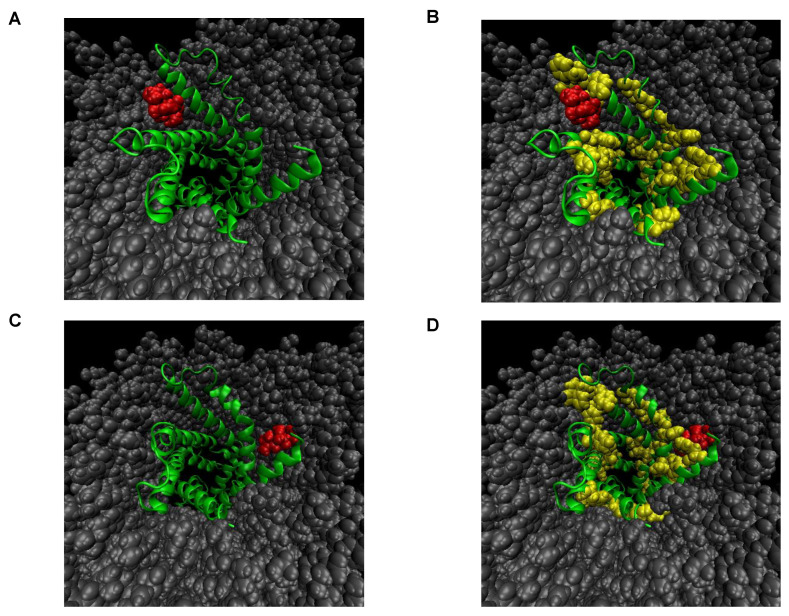
Molecular binding models for the interaction between rabdosianone I with ANT2 in the membrane. (**A**) Most populated complex structure of rabdosianone I with ANT2. (**B**) Emphasis of aromatic residues in **A**. (**C**) Secondary populated complex structure of rabdosianone I with ANT2. (**D**) Emphasis of aromatic residues in **C**. The structure of bovine mitochondrial ADP/ATP carrier (PDBcode:1OKC) with high sequence identity (90.07%) was used as a template for homology modeling. The Figures were made by PyMOL (The PyMOL Molecular Graphics System, Version 2.0 Schrödinger, LLC.). Color shows ANT2 (green), aromatic residues (yellow), rabdosianone I (red), and POPC lipid (gray).

The microscopic state of the interaction between rabdosianone I and its binding proteins was studied by molecular modeling and molecular dynamics (MD) simulation in a water/membrane environment. As the structure of ANT2 is not solved experimentally, we tried to make homology modeling using the structure of the protein with a similar sequence to the target protein. We then used the structure of bovine mitochondrial ADP/ATP carrier (PDBcode:1OKC) with high sequence identity (90.07%) as a template for homology modeling. Next, we generated 1200 complex structures between ANT2 and rabdosianone I, whose atomic information is shown in Supplementary Table S1, by docking simulation. These analyzed structures were classified as two conformations (Figure 4A–D). In both complex structures, rabdosianone I was located near the hydrophobic cleft, which, of note, consists of an aromatic amino acid (phenylalanine, tyrosine, and tryptophan) indicating that the binding mode is stabilized by not only hydrophobic interaction but also π-π stacking. Furthermore, to investigate the stability of the complex structure, we carried out MD simulation. As ANT2 is a mitochondrial membrane protein, a water solvated membrane (POPC) box was prepared, and the rabdosianone I-ANT2 complex was put into the center of the membrane. The system was simulated for 10 ns. In all simulations, rabdosianone I kept the position on the hydrophobic pocket of ANT2. We then calculated the difference between potential binding energies in bound and unbound states (ΔE = E_bound_ − E_unbound_) for two conformations. The value of binding energy was −13.4 kcal/mol for one conformation shown in Figure 4A and −8.1 kcal/mol for the other conformation shown in Figure 4C. These results suggest that the interaction between rabdosianone I and ANT2 is quite strong (Supplementary Video S1A,B). 

### 2.5. The Depletion of ANT2 or PHB2 Reduces TS Expression

**Figure 5:**
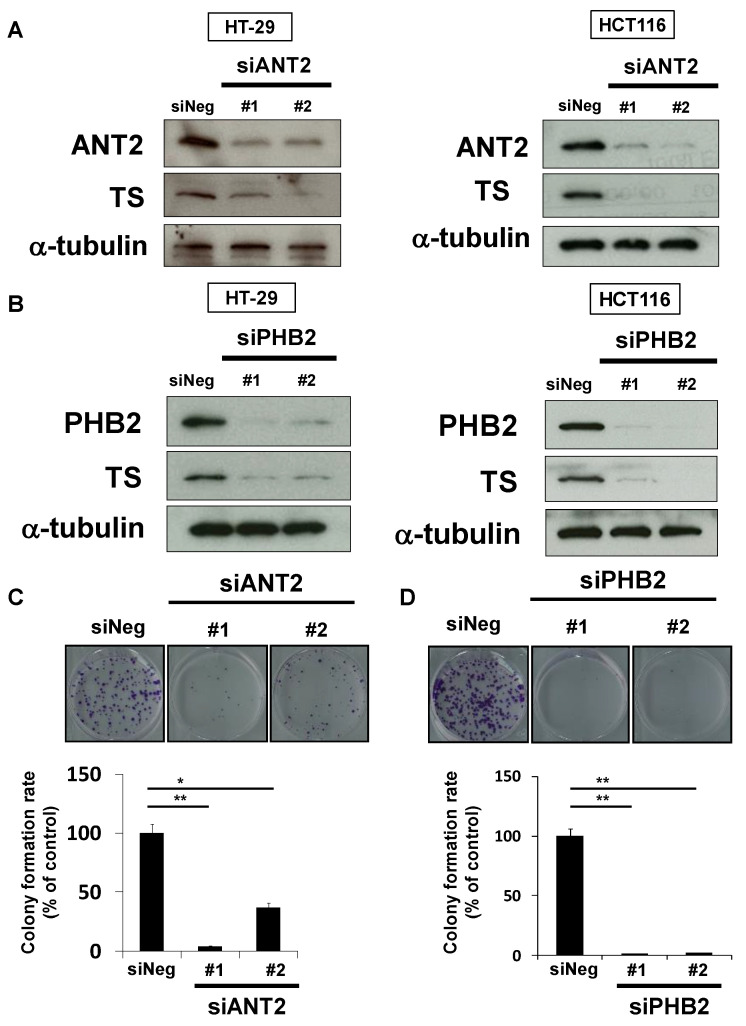
Depletion of ANT2 or PHB2 reduces TS expression and suppresses cell growth. (**A**) Effects of ANT2 depletion on TS protein expression. TS was analyzed by Western blotting in cells treated with siRNAs (#1 and #2) targeting different sequences of ANT2 gene or siNeg for 48 h (HT-29 cells, left panel) or 72 h (HCT116 cells, right panel). α-Tubulin was used as a loading control. (**B**) Effects of PHB2 depletion on TS protein expression. TS was analyzed by Western blotting in cells treated with siRNAs (#1 and #2) targeting different sequences of PHB2 gene or siNeg for 48 h (HT-29 cells, left panel) or 72 h (HCT116 cells, right panel). α-Tubulin was used as a loading control. (**C**) Effects of ANT2 depletion on colony formation. HT-29 cells were treated with siANT2 or siNeg. After further incubation, colonies were fixed and stained with crystal violet. The representative images of stained colonies are shown (upper panel). Colony formation rates are shown in the graph (lower panel). Columns, means (*n* = 3); bars, SD, * *p* < 0.05, ** *p* < 0.01, significantly different from the siNeg-treated control. (**D**) Effects of PHB2 depletion on colony formation. HT-29 cells were treated with siPHB2 or siNeg. After further incubation, colonies were fixed and stained by crystal violet. The representative images of stained colonies are shown (upper panel). Colony formation rates are shown in the graph (lower panel). Columns, means (*n* = 3); bars, SD, ** *p* < 0.01, significantly different from the siNeg-treated control.

We next analyzed whether rabdosianone I-bound ANT2 or PHB2 affects TS expression using siRNAs targeting different sequences of each gene. The depletion of ANT2 reduced TS expression in HT-29 and HCT116 cells (Figure 5A). Similarly, the depletion of PHB2 also reduced TS expression in both cell lines (Figure 5B). Moreover, treatment with siANT2 or siPHB2 significantly suppressed colony formation by HT-29 cells (Figure 5C,D). These results suggest that ANT2 and PHB2 positively regulate TS expression, and rabdosianone I binding to ANT2 and PHB2 suppresses their functions.

### 2.6. ANT2 and PHB2 May Prevent Proteasomal Degradation of TS Protein, Whereas PHB2 May also Increase TS mRNA Expression

**Figure 6:**
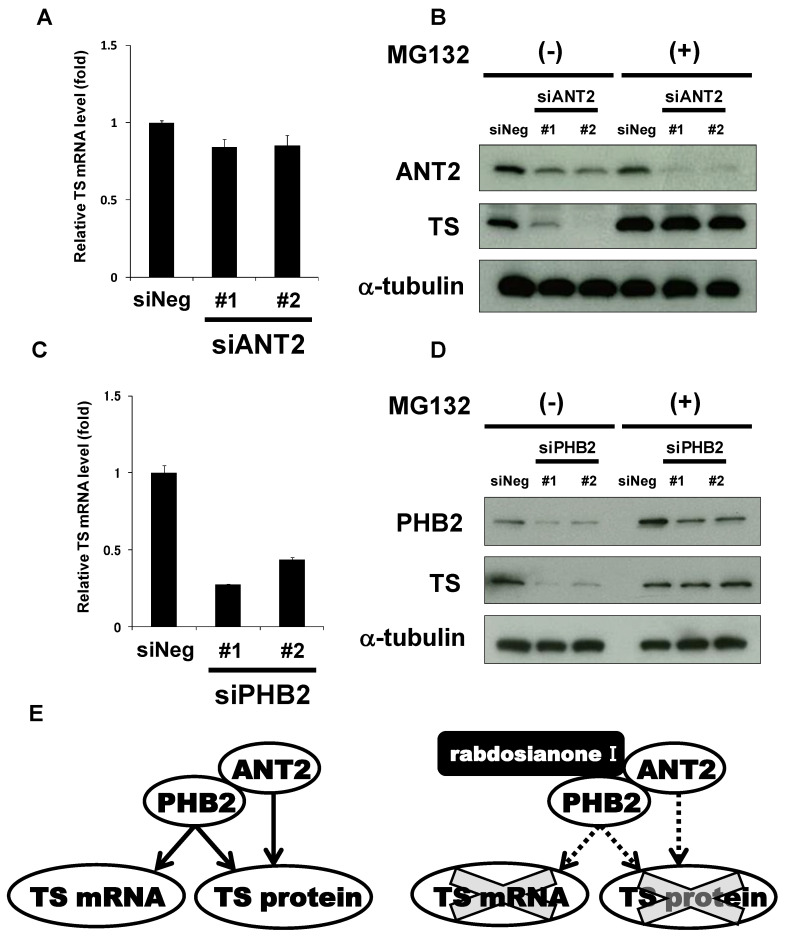
ANT2 and PHB2 may prevent proteasomal degradation of TS protein, whereas PHB2 may also increase TS mRNA expression. (**A**) Effects of ANT2 depletion on TS mRNA expression. The expression of TS mRNA was measured by quantitative PCR in HT-29 cells treated with siANT2 or siNeg for 48 h. TS mRNA was normalized to GAPDH mRNA, and the results with siNeg were taken as 1. Columns, means (*n* = 3); bars, SD. (**B**) Effects of ANT2 depletion on TS protein expression with or without MG132 treatment. HT-29 cells were treated with siANT2 or siNeg for 24 h, and the medium was then replaced with that containing 10 μM MG132 or DMSO. After incubation for 24 h, ANT2 and TS were analyzed by Western blotting. α-Tubulin was used as a loading control. (**C**) Effects of PHB2 depletion on TS mRNA expression. The expression of TS mRNA was measured by quantitative PCR in HT-29 cells treated with siPHB2 or siNeg for 48 h. TS mRNA was normalized to GAPDH mRNA, and the results with siNeg were taken as 1. Columns, means (*n* = 3); bars, SD. (**D**) Effects of PHB2 depletion on TS protein expression with or without MG132. HT-29 cells were treated with siPHB2 or siNeg for 24 h, and the medium was then replaced with that containing 10 μM MG132 or DMSO. After incubation for 24 h, PHB2 and TS were analyzed by Western blotting. α-Tubulin was used as a loading control. (**E**) Schematic representation of the pleiotropic regulation of TS by ANT2 and PHB2. ANT2 and PHB2 may stabilize TS at the protein level, whereas PHB2 may also increase TS mRNA expression (e.g., promoting transcription). Rabdosianone I binding to ANT2 and PHB2 may promote proteasomal degradation of TS protein, whereas its binding to PHB2 may suppress TS transcription.

To further examine how ANT2 regulates TS expression, we first analyzed TS mRNA levels in siANT2-treated cells. Depletion of ANT2 did not alter the expression levels of TS mRNA (Figure 6A). On the other hand, siANT2-mediated TS reduction was inhibited by the addition of MG132 (Figure 6B), suggesting that ANT2 prevents proteasomal degradation of TS protein. On the other hand, depletion of PHB2 significantly reduced the expression of TS mRNA (Figure 6C). Furthermore, siPHB2-mediated TS reduction was restored by the addition of MG132 (Figure 6D). These results suggest that TS expression is regulated by two distinct pathways, ANT2 and PHB2-mediated protein stabilization and PHB2-mediated increase in mRNA level.

## 3. Discussion

In this study, our chemical biology approach led to the discovery of pleiotropic regulatory mechanisms of the TS oncogene expression through ANT2 and PHB2, which are binding proteins of the oriental ancient herb extract rabdosianone I. Rabdosianone I promoted the proteasomal degradation of TS protein by directly binding to ANT2 and PHB2, which may stabilize TS protein. It also suppressed the expression of TS mRNA by directly binding to PHB2, which may promote the transcription of TS gene or stabilize TS mRNA (Figure 6E). 

Our unique approach presented in this study was the operation of MD simulation. Chemical biology method in this study is a pull-down assay using magnetic beads, which is undeniable to be an “artificial experiment”. To compensate this limitation, we conducted MD simulation to help in better understanding of the physiological binding mode of rabdosianone I with ANT2 in the mitochondrial membrane (Figure 4A–D and Supplementary Video S1A,B). Thus, although MD simulation is just a simulation, it can predict the precise binding site and mode with calculation of the binding energy. Interestingly, we previously reported that sesaminol, a sesame lignan, also binds to ANT2 [24]; however, the structures of rabdosianone I and sesaminol are quite different from each other. Thus, it appears that the affinity between the compound and the molecule could sometimes not be explained by the structure-activity relationship, and in such cases, MD simulation could be a useful tool and may have an advantage to broadly screen the compound binding to the specific protein. 

The degradation of TS protein is facilitated by unique machinery that is dependent on the 26 S proteasome, similar to many other proteins, but not on polyubiquitination [25,26]. Instead of polyubiquitination of lysine residues, the N-terminal region of TS protein, which does not affect its catalytic activity, acts as a proteasomal degradation signal [27,28]. We demonstrated that rabdosianone I treatment or the depletion of ANT2 and PHB2 promoted the proteasomal degradation of TS protein by experiments using the proteasomal inhibitor MG132. Recently, ANT2 shRNA was reported to suppress the activity of a chaperone protein HSP90 and promote degradation of its client protein HER2 [29]. As another example, we previously reported that ANT2 can protect cyclin D1 protein from proteasomal degradation [24]. Thus, ANT2 may function in the stability of some proteins, but the mechanism(s) involved in this process should be investigated further.

Regarding the regulation of TS at the mRNA level, E2F1-dependent transcription of the TS gene is well known [30,31,32]. Of note, PHB2 located outside of the mitochondria may promote E2F1-dependent TS expression. That is, PHB2 is also located in membrane lipid rafts, and forms a complex with RAS and CRaf, leading to the activation of the MAPK cascade [33,34]. Considering that the inhibition of the MAPK cascade results in the activation of RB protein and the suppression of the transcriptional activity of E2F1 [35,36], rabdosianone I-bound PHB2 in the plasma membrane may suppress TS expression by blocking the MAPK cascade. Thus, the subcellular localization of PHB2-bound rabdosianone I should be further examined to clarify the complete mechanism(s) of PHB2-mediated regulation of TS mRNA.

There are still other problems to be considered. First, while rabdosianone I sufficiently suppressed TS expression at 4–8 μM, it showed the growth inhibitory effect under these concentrations. Thus, it is reasonable to consider that other molecules other than TS are also involved in rabdosianone I-mediated growth inhibition. Second, in HCT-116 cells, 8 μM rabdosianone I recovered S arrest (Figure 1G, right panel) and TS expression (Figure 2A, right panel), which implies the involvement of some sort of the feedback system. As an example, TS binds to its own mRNA, resulting in the inhibition of its translation [5,37]. Thus, under some circumstances, rabdosianone I-mediated TS suppression may lead to TS induction by such a feedback reaction, although how and when this paradoxical phenomenon is observed needs to be elucidated in future studies. Lastly, in this study, we first found that ANT2 and PHB2 act upstream of TS; however, more work is required to unravel whether and how ANT2 and PHB2 are expressed and regulated in specific cancer tissues.

## 4. Materials and Methods 

### 4.1. Reagents

Rabdosianone I was kindly provided by Dr. Yasumasa Yamada (Baika Women’s University). The purity of rabdosianone I was confirmed to be approximately 98% by LCMS, including LC chromatogram and TOF-MS. MG132 was obtained from CALBIOCHEM (San Diego, CA, USA). These reagents were dissolved in the solvent dimethyl sulfoxide (DMSO) to make stock solutions. Purified recombinant human ANT2 (Homo sapiens solute carrier family 25, member 5, catalog No: TP308949) and PHB2 (Human prohibitin 2, catalog No: TP760501) were obtained from OriGene (Rockville, MD, USA). 

### 4.2. Cell Lines and Culture

The human colorectal cancer cell lines HT-29, HCT116, and HCT-15 were obtained as NCI-60 cell lines from the NCI Developmental Therapeutics Program. The human colorectal cancer cell line SW480 and normal breast epithelial cell line MCF-10A were obtained from the American Type Culture Collection. Mouse colon mucosa cells were established and kindly provided by Dr. Shingo Miyamoto (National Cancer Center). The authenticity of each commercially available cell line was confirmed by short tandem repeat profiling at the cell bank. All cell lines were confirmed to be negative for mycoplasma infection using the MycoAlertTM Mycoplasma Detection Kit (Lonza, Rockland, ME, USA). All cell lines except for HCT-15 and mouse colon mucosa cells were cultured in Dulbecco’s modified Eagle’s medium (DMEM) supplemented with 10% fetal bovine serum (FBS), 4 mM glutamine, 50 U/mL penicillin, and 100 μg/mL streptomycin. HCT-15 cells were cultured in RPMI-1640 supplemented with 10% FBS, 4 mM glutamine, 50 U/mL penicillin and 100 μg/mL streptomycin. Mouse colon mucosa cells were cultured in the maintaining medium containing penicillin/streptomycin, HEPES, Glutamax, N2 Supplement, B27 Supplement (all from Life Technologies, Carlsbad, CA, USA), N-acetylcysteine, gastrin, nicotinamide, SB202190 (all from Sigma, St Louis, MO, USA), EGF, dorsomorphin, Y-27632, A-83-01 (all from Wako Chemicals, Osaka, Japan), GSK-3 inhibitor XV (Santa Cruz Biotechnology, Dallas, TX, USA), CHIR99021 (Axon Medchem, Groningen, Netherlands), and 2% FBS in advanced DMEM/F12 (Life Technologies). Cells were incubated at 37 °C in a humidified atmosphere of 5% CO_2_.

### 4.3. Cell Viability Assay

The number of viable cells was measured by a Cell Counting Kit-8 assay (Dojindo, Kumamoto, Japan) according to the manufacturer’s instructions as previously described [38]. Briefly, after cells were seeded in 96-well plates at a density of 2000 cells (HT-29, HCT116, SW480, and HCT-15) or 5000–10,000 cells (mouse colon mucosa cells and MCF-10A) per well and incubated for 24 h, cells were treated with the agent or siRNAs, and then the kit reagent WST-8 was added to the medium and incubated for 4 h. The absorbance at 450 nm of the samples was measured using a multi-plate reader (Molecular Devices, LLC., San Jose, CA, USA).

### 4.4. Colony Formation Assay

Colony formation assays were performed as previously described [39]. Briefly, after cells were seeded in 6-well plates at a density of 200 cells and incubated for 24 h, cells were treated with the agent or siRNAs. After further incubation for approximately 14 days, the cells were fixed with 10% formalin and stained with 0.1% crystal violet. The area of stained colonies was quantified using the ImageJ program from the National Institutes of Health (Bethesda, MD, USA, https://imagej.nih.gov/ij/ (accessed on 28 August 2015).

### 4.5. Cell Cycle Analysis and Detection of Apoptosis

Cell cycle and apoptosis induction were analyzed as previously described [40]. Briefly, after cells were seeded in 6-well plates at a density of 50,000 cells per well and incubated for 24 h, the cells were treated with the agent or siRNAs and harvested by trypsinization. After centrifugation, cells were suspended in PBS containing 0.1% Triton X-100 and 25 μg/mL propidium iodide. Stained cells were analyzed using FACSCalibur (Becton Dickinson, Franklin Lakes, NJ, USA). Cell cycles were analyzed using Modfit LT software (Becton Dickinson). As for the detection of apoptosis, Cell Quest software (Becton Dickinson) was used to quantify the percentage of hypodiploid DNA as the sub-G1 population.

### 4.6. Protein Isolation and Western Blotting

Protein isolation was performed as previously described [41]. Briefly, cells were lysed with lysis buffer containing 50 mM Tris-HCl, 1% SDS, 1 mM dithiothreitol (DTT), and 0.43 mM 4-(2-Aminoethyl) benzenesulfonyl fluoride hydrochloride (ABSF). The lysates were sonicated and centrifuged at 20,400× *g* at 4 °C for 20 min, and the supernatants were collected. Equal amounts of protein extract were subjected to SDS-PAGE and transferred to a PVDF (polyvinylidene difluoride) membrane (EMD Millipore, Billerica, MA, USA). The following were used as the primary antibodies: Mouse anti-human thymidylate synthase monoclonal antibody (ab58287; Abcam, Cambridge, UK), mouse anti-human β-actin monoclonal antibody (A5441; Sigma), mouse anti-human α-tubulin monoclonal antibody (CP06; CALBIOCHEM), mouse anti-human ANT2 polyclonal antibody (H00000292-B01P; Novus Biologicals, LLC, Centennial, CO, USA), rabbit anti-human PHB2 monoclonal antibody (#14085; Cell Signaling Technology, Danvers, MA, USA), mouse anti-DDK(FLAG) monoclonal antibody (TA50011-100; Origene), and mouse anti-His-Tag monoclonal antibody (#2366; Cell Signaling Technology). Signals were detected with Chemi-Lumi One L (Nacalai Tesque, Kyoto, Japan) or ImmobilonTM Western Chemiluminescent HRP Substrate (EMD Millipore).

### 4.7. RNA Isolation and Quantitative PCR

RNA isolation and quantitative PCR were performed as previously described [42]. Briefly, total RNA was isolated from cells treated with the agent or siRNAs using Sepasol-RNA I (Nacalai Tesque) according to the manufacturer’s instructions. Total RNA (2 μg) was reverse-transcribed to complementary DNA (cDNA) in a 20 μL reaction volume with MMTV-reverse transcriptase (Promega) and oligo (dT) primers (Toyobo, Osaka, Japan). An equivalent volume of cDNA solution was used for quantitative PCR. cDNA was amplified using an ABI 7300 real-time PCR system (Applied Biosystems, Foster City, CA, USA) with TaqMan Probes for TYMS (Hs00426591_m1) and GAPDH (Hs02758991_g1) (Applied Biosystems). The expression of thymidylate synthase mRNA was normalized to that of GAPDH mRNA in the same sample.

### 4.8. RNAi

RNAi was performed as previously described [43]. Briefly, oligonucleotides of siRNA for thymidylate synthase, ANT2, and PHB2 were obtained from Dharmacon (Lafayette, CO, USA). The following siRNAs were used: siTS #1 (J-004717-07; ON-TARGET plus Human TYMS (7298) siRNA), 5′-UGGGAGAUGCACAUAUUUA-3′; siTS #2 (J-004717-08; ON-TARGET plus Human TYMS (7298) siRNA), 5′-UCACAUCGAGCCACUGAAA-3′; siANT2 #1 (D-007486-03; siGENOME Human SLC25A5 (292) siRNA), 5′-CUGCAGAUAAGCAAUACAA-3′; siANT2 #2 (D-007486-04; siGENOME Human SLC25A5 (292) siRNA), 5′-GCAGAUAAGCAAUACAAAG-3′; siPHB2 #1 (J-018703-07; ON-TARGET plus Human PHB2 (11331) siRNA), 5′-CAUCAAACUUCGCAAGAUU-3′; siPHB2 #2 (J-018703-08; ON-TARGET plus Human PHB2 (11331) siRNA), 5′-CAGAAUAUCUCCAAGACGA-3′. The following negative control siRNAs were used: ON-TARGET plus Non-Targeting siRNA #3 (D-001810-03), 5′-UGGUUUACAUGUUUUCUGA-3′; siGENOME Non-Targeting siRNA #5 (D-001210-05), 5′-UGGUUUACAUGUCGACUAA-3′. Cells were transfected with 10 nM siTS and siPHB2 or 30 nM siANT2 using Lipofectamine RNAiMAX Reagent (Invitrogen, Carlsbad, CA, USA) according to the manufacturer’s instructions.

### 4.9. Preparation of Rabdosianone I-Fixed Beads

Magnetic FG beads with epoxy linkers were purchased from Tamagawa Seiki (Nagano, Japan). The beads were incubated with 100 mM rabdosianone I in DMF containing potassium carbonate at 60 °C for 24 h as previously described [24]. After being washed twice with DMF, the beads were then washed with Milli-Q water.

### 4.10. Purification and Identification of Rabdosianone I-Binding Proteins

Purification and identification of rabdosianone I-binding proteins were performed as previously described [24]. Briefly, HT-29 cells were lysed with binding buffer containing 50 mM Tris-HCl, 150 mM NaCl, 1% NP-40, 1 mM DTT, and 0.43 mM ABSF at 4 °C for 30 min, and centrifuged. The supernatants were used as whole-cell extracts of HT-29 cells. The extracts were incubated with the agent-fixed beads or empty beads at 4 °C for 4 h. The beads were washed 3 times with binding buffer. The bound proteins were eluted with Laemmli dye and subjected to SDS-PAGE. The proteins were stained by aqueous AgNO_3_, and each strip, including the protein, was cut off to apply Sequencing Grade Modified Trypsin (Promega, Madison, WI, USA). The peptide fragments from each strip were analyzed using an Autoflex II mass spectrometer (Bruker Daltonics, Billerica, MA, USA) after in-gel digestion.

### 4.11. Molecular Modeling and Docking Simulation

The structure of ANT2 was generated by homology modeling server SWISSMODEL [44]. The structure of bovine mitochondrial ADP/ATP carrier (PDBcode:1OKC) was used as a template for homology modeling. Docking simulation between ANT2 and rabdosianone I was carried out by zdock 3.0.2 [45]. The Figures were made by PyMOL (The PyMOL Molecular Graphics System, Version 2.0 Schrödinger, LLC.).

### 4.12. Molecular Dynamics Simulation

The microscopic state of interactions between rabdosianone I and ANT2 protein were studied by molecular modeling and molecular dynamics (MD) simulation in a water/membrane environment. ANT2 protein was described using the CHARMM36 force field [46]. Rabdosianone I was also described using the CHARMM36 force field with restrained electrostatic potential (RESP) charges [47]. The partial charge of rabdosianone I is presented in Supplementary Table S1. Water molecules were described using the TIP3P model [48]. The system contained a protein, 149 lipid molecules, 12,541 water molecules, and 33 sodium and chloride ions corresponding to 150 mM solution. To neutralize the net charge of the system, 15 chloride ions were added. These molecules were placed in a rectangular box with (x, y, z) = (77.4 Å, 77.4 Å, 99.9 Å). The temperature was maintained at 300 K using Langevin dynamics, and a pressure of 1 atm was maintained using Parinello-Rahman barostat [49]. The MD simulations were conducted using the GROMACS 2016 simulator [50]. The Figures were made by VMD [51].

### 4.13. Statistical Analysis

All quantitative data were presented as the mean ± standard deviation (SD). The significance of differences in the means among 3 or more groups was assessed using a one-way ANOVA, and that of comparisons between two groups was evaluated using a two-tailed unpaired Student’s *t*-test. A value of *p* < 0.05 was considered to indicate a significant difference from each control.

## 5. Conclusions

Our chemical biology approach revealed novel anticancer mechanisms of rabdosianone I, an extract from the traditional Asian herb *Isodon japonicus* Hara for longevity. Rabdosianone I directly targeted two mitochondrial inner proteins ANT2 and PHB2, resulting in suppression of TS at multiple levels, including mRNA expression and protein stability. Thus, our study not only led to the discovery of the pleiotropic signaling of TS regulation by ANT2 and PHB2 but also constitutes a starting point for the design of novel TS targeting agents based on rabdosianone I analogs to provide opportunities for cancer treatment and prevention.

## Data Availability

The data presented in this study are available on reasonable request to the corresponding author.

## Supplementary Materials

The following are available online at https://www.mdpi.com/2072-6694/13/5/982/s1, Figure S1: The purity analyses of rabdosianone I, Figure S2: Sub G1 population following rabdosianone I treatment, Figure S3: The effects of rabdosianone I on the expression levels of ANT2 and PHB2, Table S1: The partial charge and atom type of rabdosianone I in mol2 format, Video S1: Molecular dynamics simulation of the complex structure of rabdosianone I with ANT2 in POPC membrane.
