# Supplementary material for: Rabdosianone I, a Bitter Diterpene from an Oriental Herb, Suppresses Thymidylate Synthase Expression by Directly Binding to ANT2 and PHB2
Source: Cancers (Basel). 2021 Feb 26;13(5):982. doi: 10.3390/cancers13050982 (PMC7956614; doi:10.3390/cancers13050982)
Supplement: Supplementary file 1 [file cancers-13-00982-s001.zip › cancers-1103160/supplementary.docx]

Supplementary Materials:

Rabdosianone I, a bitter diterpene from an oriental herb, suppresses thymidylate synthase expression by directly binding to ANT2 and PHB2

Motoki Watanabe ^1,^*, Yasumasa Yamada ^2^, Yoichi Kurumida ^3^, Tomoshi Kameda ^3^, Mamiko Sukeno ^4^, Mahiro Iizuka-Ohashi ^1,5^, Yoshihiro Sowa ^1^, Yosuke Iizumi ^1^, Hideki Takakura ^1^, Shingo Miyamoto ^6^, Toshiyuki Sakai ^4^ and Michihiro Mutoh ^1,6^

**A**

**B**

**Figure S1.** The purity analyses of rabdosianone I. (**A**) LC chromatogram of rabdosianone I. (**B**) Mass fragmentation pattern of tailing moiety (upper panel) and sharp peak (lower panel) by TOF-MS (LCMS).

**Figure S2.** Sub G1 population following rabdosianone I treatment. HT-29 and HCT116 cells were treated with rabdosianone I at the indicated concentrations for 72 hr. The percentages of cells in the sub G1 population were analyzed by flow cytometry. Columns, means (n = 3); bars, SD. **P<0.01, significantly different from the DMSO-treated control.

**A B**

**Figure S3.** The effects of rabdosianone I on the expression levels of ANT2 and PHB2. (**A,B**) ANT2 and PHB2 protein expressions in rabdosianone I-treated cells. The protein expressions of ANT2 (**A**) and PHB2 (**B**) were analyzed by Western blotting in HT-29 cells treated with rabdosianone I at the indicated concentrations for 48 hr. β-Actin and α-tubulin were used as a loading control.

 **Continued on the following page.**

**Continued from the previous page.**

**Table S1.** The partial charge and atom type of rabdosianone I in mol2 format.

**Video S1.** Molecular dynamics simulation of the complex structure of rabdosianone I with ANT2 in POPC membrane. (**A,B**) The movies show 10 ns simulation from two initial structures generated by docking simulation. Color shows ANT2 (green), aromatic residues (yellow), POPC (gray) and rabdosianone I (red).
